# Recurrent Orbital Metastases in Follicular Variant Papillary Thyroid Carcinoma After Recombinant Human Thyrotropin

**DOI:** 10.1210/jcemcr/luaf320

**Published:** 2026-01-14

**Authors:** Eiman Ibrahim, Lilamani Kurukulasuriya, Tabitha Galloway, Mohamed Elbanan, Muhammad Moseeb Ali Hashim, Michael Gardner

**Affiliations:** Department of Endocrinology, University of Missouri School of Medicine, Columbia, MO 65201, USA; Department of Endocrinology, University of Missouri School of Medicine, Columbia, MO 65201, USA; Department of Otolaryngology, University of Missouri School of Medicine, Columbia, MO 65201, USA; Department of Diagnostic Radiology, University of Missouri School of Medicine, Columbia, MO 65201, USA; Department of Pathology, University of Missouri School of Medicine, Columbia, MO 65201, USA; Department of Endocrinology, University of Missouri School of Medicine, Columbia, MO 65201, USA

**Keywords:** papillary thyroid carcinoma, orbital metastases, recombinant human thyrotropin, follicular variant papillary thyroid carcinoma

## Abstract

Recombinant human thyrotropin (rhTSH) is generally well tolerated with minimal adverse effects. Symptomatic tumor expansion following rhTSH has been previously reported in the literature. Generally, these cases have involved known metastasis of aggressive variants. In this report we present a case of follicular variant papillary thyroid carcinoma (FVPTC) with recurrent treated orbital metastases and adverse response to rhTSH. Following a second dose of rhTSH, the patient reported right eye proptosis, and edema was noted on imaging. Iodine-131 scan demonstrated intense right orbital uptake with intracranial extension verified on head scans. Biopsy confirmed FVPTC and she underwent orbitotomy and enucleation. Our case illustrates the potential risk of symptomatic expansion even in cases of previously treated metastasis.

## Introduction

Differentiated thyroid cancer (DTC) is currently one of the most rapidly increasing cancers. Annual incidence rates approximate 1 in 10 000 individuals with a 3:1 female-to-male ratio. Papillary and follicular variants of DTC constitute the majority of these diagnoses [1]. Generally, DTC often progresses slowly and has an excellent prognosis, with 10-year survival rates exceeding 90% [2].

The follicular variant of papillary thyroid carcinoma (FVPTC) is recognized as the second most prevalent subtype of papillary thyroid carcinoma (PTC), following the classic type. It accounts for approximately 20% of all PTC cases [3]. FVPTC is typically a slow-growing and a clinically indolent tumor, but it may become invasive, extending locally, and causing distant metastases in some situations [4]. Histopathologically, FVPTC consists of a follicular architectural pattern with nuclear features of PTC [4]. It can form multiple tumor foci within the thyroid gland and spreads via lymphatic or hematogenous routes [5].

Thyroid carcinomas metastasizing to the choroids and orbits are rare. A systematic review of case reports from 1977 to 2012 identified only 31 cases of orbital and/or ocular metastases from thyroid cancer [6]. Most patients with these metastases had a long-standing history of thyroid cancer and widespread metastatic disease. PTC was the most common primary tumor, followed by medullary and follicular thyroid carcinoma [6]. Diagnosis typically relied on noninvasive techniques. Iodine-131 (I-131) scanning showed uptake in the orbit in a subset of the described cases [6, 7].

Although orbital metastases from thyroid carcinomas are rare, thyroid carcinoma should be considered as a potential primary tumor in patients with orbital metastasis [8].

Standard treatment for patients with distantly metastatic DTC typically involves a thyroidectomy or thyroid lobectomy, central or lateral compartment lymphadenectomy if indicated, followed by radioiodine (RAI) therapy [9].

According to the recent joint statement from the American Thyroid Association, the European Association of Nuclear Medicine and Molecular Imaging, the Society of Nuclear Medicine and Molecular Imaging, and the European Thyroid Association [10], the primary objectives of RAI administration should be goal-oriented and categorized into 3 primary objectives: remnant ablation, adjuvant therapy, and treatment of known disease. Remnant ablation aims to eradicate presumably benign residual thyroid tissue following total thyroidectomy, thereby optimizing serum thyroglobulin interpretation and enhancing the quality of future I-131 imaging. In contrast, adjuvant therapy is designed to reduce the risk of recurrence by targeting potential microscopic foci of thyroid cancer tissue after complete surgical resection of metastatic disease. Additionally, RAI therapy is also used to address known persistent disease, offering a comprehensive approach to managing DTC [10].

The administration of RAI necessitates thyrotropin (TSH) stimulation, achievable through 2 methods [1]: withdrawal of levothyroxine (L-T4) to induce endogenous TSH elevation, or [2] exogenous stimulation using recombinant human thyrotropin (rhTSH) [11]. The rhTSH glycoprotein is produced by transfecting a genetically modified Chinese hamster ovary cell line with plasmids containing the α and β sequences of TSH [11, 12]. It features lower glycosylation and higher sialylation levels than endogenous TSH, resulting in 3 to 4 times lower TSH receptor affinity but a longer half-life.

The suggested standard dose of rhTSH is 0.9 mg administered intramuscularly over 2 consecutive days [11, 13]. After injection, peak TSH concentrations are typically reached within 10 hours [11, 14]. There is considerable individual variability in serum TSH levels achieved after rhTSH administration, influenced by factors such as age, body surface area, and body mass index [15]. In contrast, endogenous TSH stimulation via L-T4 withdrawal results in a less intense but more sustained TSH elevation [14, 16].

Patients treated with rhTSH retain a satisfactory quality of life without experiencing the symptoms of hypothyroidism [12, 17]. This is particularly advantageous for individuals with brain or spinal metastases, as it helps avoid chronic TSH stimulation that could lead to tumor growth. Conversely, the peak TSH levels following rhTSH administration are notably greater than those observed after withdrawal, which may lead to an elevated risk of some adverse effects, such as hemiplegia and bone pain at metastatic sites, which also have been reported [17]. The European Medicines Agency approved rhTSH in 2005, and the US Food and Drug Administration followed in 2007 for specific indications, allowing it to prevent symptomatic hypothyroidism, which can be particularly challenging for older patients and those with additional health issues [18]. Moreover, rhTSH stimulation improves imaging sensitivity in patients with high thyroglobulin, particularly on 18F-fluorodeoxyglucose (^18^F-FDG) positron emission tomography (PET) scanning with an expected absolute increase of approximately 14% [19].

Here, we report a patient who underwent a typical management approach for suspected recurrence of DTC, which resulted in the unanticipated detection of orbital metastatic recurrence.

## Case Presentation

**Figure 1:**
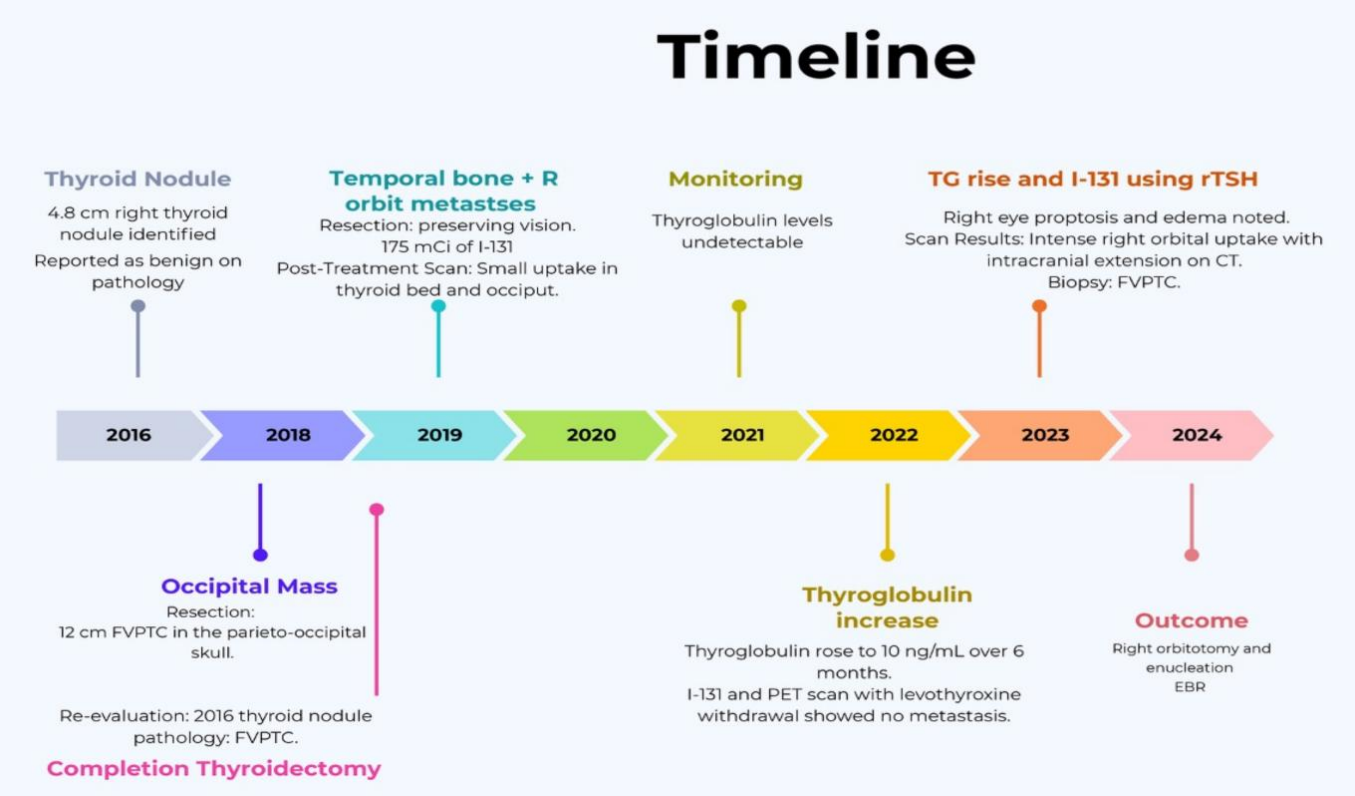
Overview of the sequence of clinical presentation, diagnostic evaluations, and therapeutic interventions over time.

In early 2018, a 70-year-old woman with a past medical history of congenital optic atrophy presented to the endocrinology office after removal of a 12-cm parieto-occipital scalp mass. Pathological analysis revealed this to be a site of metastatic FVPTC (Fig 1**)**.

At the time of initial detection of metastasis, her serum thyroglobulin levels were markedly elevated at 778 ng/mL (SI: 778 µg/L) (reference range, <0.2-0.4 ng/mL [SI: <0.2-0.4 μg/L]), consistent with active metastatic disease. Following surgical resection of the scalp mass, her thyroglobulin levels demonstrated a significant decline to less than 0.1 ng/mL (SI: <0.1 µg/L) (reference range, <0.2-0.4 ng/mL [SI: <0.2-0.4 μg/L]), reflecting a reduction in tumor burden.

The patient 2 years prior had undergone a right thyroidectomy for a 4.8-cm right thyroid nodule. Final pathology at the time of the procedure was reported at an outside institution as benign. On subsequent re-review following craniectomy, this was amended to FVPTC.

## Diagnostic Assessment

**Figure 2:**
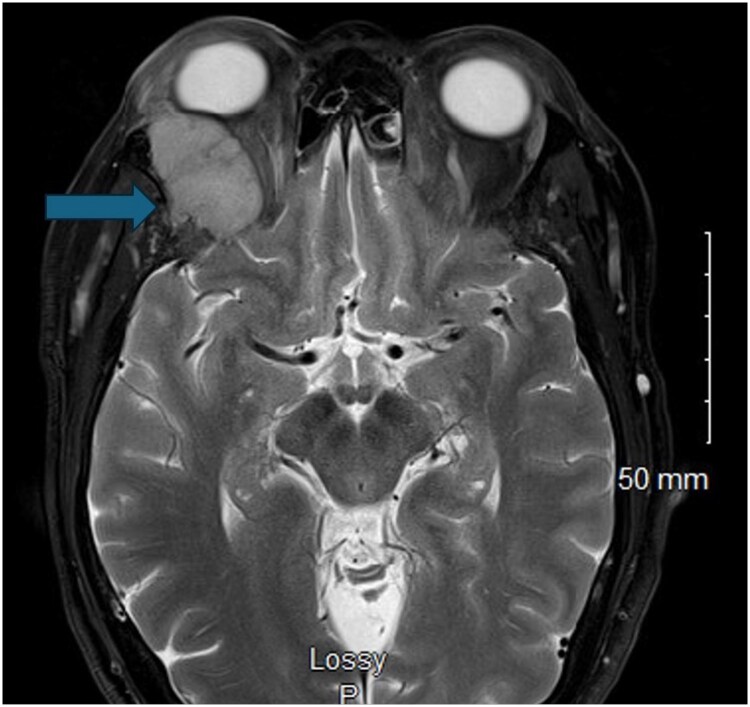
Magnetic resonance imaging of the orbit showing right lateral orbital wall mass lesion with effect on the adjacent optic nerve and increase in the right globe proptosis with increased displacement of the superior and lateral rectus muscles.

The patient then underwent completion thyroidectomy. Despite initial successful management, subsequent radiologic evaluations by computed tomography (CT) and magnetic resonance imaging (MRI) showed additional calvarial metastasis to the temporal bones and right orbit (Fig. 2). These were successfully resected, preserving her vision, and she received 175 mCi of I-131. The posttreatment scan revealed only small uptake in the thyroid bed as well as in the occipital region.

Thyroglobulin was undetectable until late 2022, indicating effective disease control at that time. Subsequently, it rose to 10 ng/mL (SI: 10 µg/L) over 6 months.

I-131 whole-body scan and ^18^F-FDG-PET scanning following L-T4 withdrawal showed no metastasis, and thyroglobulin stabilized below 10 ng/mL (SI: 10 µg/L) until mid-2024, when serum thyroglobulin concentration rose abruptly to 64 ng/mL (SI: 64 μg/L). Due to concern for possible dedifferentiation causing prior negative I-131 scanning, 18F-FDG-PET scanning, including skull and orbits, was repeated and again demonstrated no abnormalities. Five months later thyroglobulin rose to 181 ng/mL (SI: 181 μg/L), prompting I-131 scanning using rhTSH due to poor tolerance to previous L-T4 withdrawal.

The patient underwent an rhTSH protocol [20], with stimulated thyroglobulin 1338 ng/mL (SI: 1338 μg/L) (reference range, <0.2-0.4 ng/mL [SI: <0.2-0.4 μg/L]), TSH measurements of 79.3 μIU/mL (SI: 79.3 mIU/L) (reference range, 0.27-4.2 μIU/mL [SI: 0.27-4.2 mIU/L]), and thyroglobulin antibody level of less than 1.8 IU/mL (SI: <1.8 kIU/L) (reference range, <1.8 IU/mL [SI: <1.8 kIU/L]).

**Figure 3:**
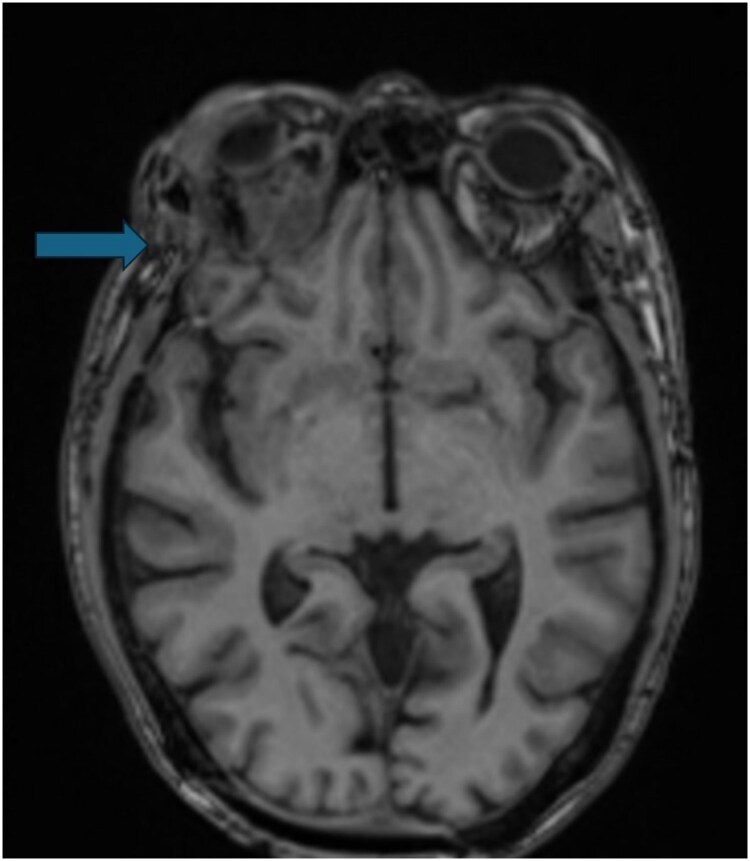
Magnetic resonance imaging of the brain showing a mass-like enhancement along the right orbital cavity with thickening/enhancement of the occipital dura and an adjacent enlarging 0.7-cm enhancing occipital calvarial metastatic lesion.

Following the second dose of rhTSH, right eye proptosis and periorbital edema were noted, and the I-131 scan demonstrated intense right orbital uptake with intracranial extension verified on CT. Immediate high-dose corticosteroids were administered without apparent benefit, and joint surgical planning was initiated. An urgent anterior orbitotomy was performed for biopsy and debulking/decompression. The specimen obtained from these again demonstrated features consistent with FVPTC. There was also a small amount of background necrosis, but no histological changes from prior specimens. The multidisciplinary tumor board recommended extirpative management with orbital enucleation and limited craniectomy due to the progression of orbital metastasis invading the intracranial space. Preoperative MRI was obtained as part of the surgical planning (Fig. 3).

## Treatment

**Figure 4:**
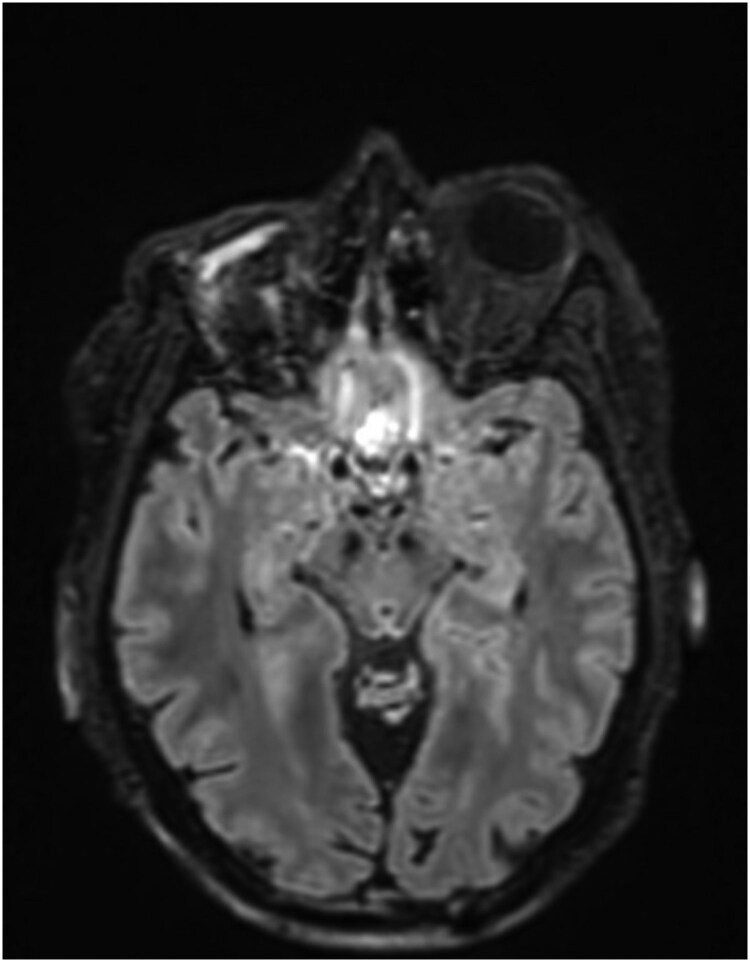
Magnetic resonance imaging of the brain showing postsurgical changes of the right orbital excision are with enhancing soft tissue mass involving the preorbital soft tissues and the floor and right lateral wall of right anterior cranial fossa.

The patient consented to this approach, prioritizing oncologic control over vision preservation in an already compromised eye. The surgery, performed in late 2024, involved collaboration between otolaryngology, ophthalmology, and neurosurgery, addressing both the orbital and intracranial components of the disease (Fig. 4**)**.

## Outcome and Follow-up

**Figure 5:**
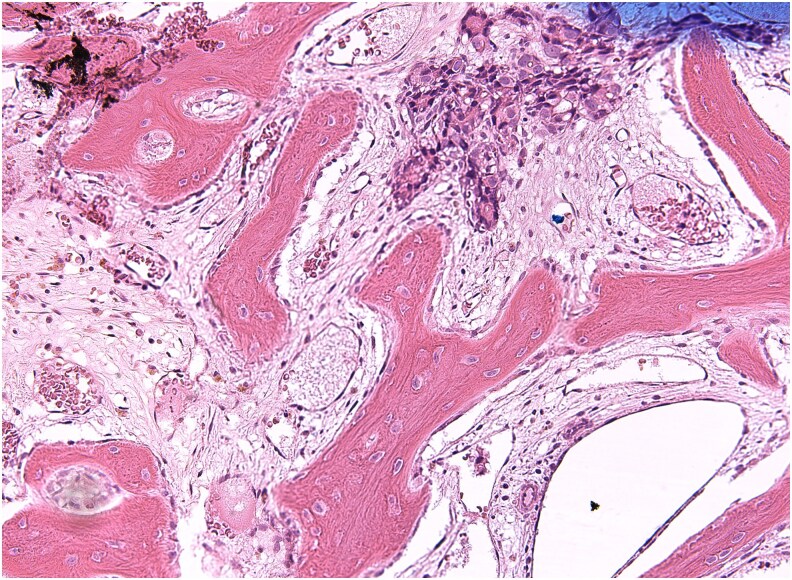
Histopathology of the resected right orbital tumor showing metastatic papillary thyroid carcinoma (microscopic magnification ×100).

The patient underwent right near-total orbital exenteration and supraorbital frontal craniotomy **(**Fig. 5). Repeat ablation and external beam radiation (EBR) were administered and well tolerated. Surgical pathology confirmed FVPTC.

## Discussion

This case demonstrates the apparent rapid enlargement of a metastatic foci of thyroid carcinoma following stimulation with rhTSH. Our patient had an aggressive FVPTC with calvarial and orbital metastases previously. However, she had responded well to initial therapy with thyroglobulin below the limits of detection for several years. Afterward, she experienced a slow return of biochemically evident disease. Despite biochemical evidence, repeated imaging, using differing modalities, demonstrated no significant tumor in the region of the orbit prior to administration of rhTSH. After rhTSH administration, she rapidly developed right eye proptosis and periorbital edema, and imaging confirmed intense orbital uptake of I-131 with an easily identifiable mass lesion, prompting extensive surgical intervention with further ablation and EBR.

Presently, there are no specific guidelines on the management of DTC with orbital metastasis aside from surgical removal of the primary tumor followed by RAI therapy ablation typical of all DTCs. Tumor expansion after rhTSH administration has been previously documented. Braga et al in 2001 [21] reported 2 patients who had PTC with locally recurrent tumors. Both experienced tumor enlargement 12 to 48 hours after receiving the second rhTSH injection, which manifested as acute respiratory distress in one patient and a palpable, tender mass in the other. Imaging techniques confirmed the tumor expansion compared to prior assessments [21]. In contrast to what is noted in the work of Braga and colleagues, our patient had no radiological or physical evidence of recurrence prior to administration of rhTSH. Given the previously indolent nature of her tumor, it is unlikely that new gross tumor developed, to the degree seen on imaging post rhTSH administration, silently in the interval between scans.

Mejia et al in 2021 [22] reported a patient with metastatic PTC who experienced rapid progression of liver and bone metastases following rhTSH administration. After rhTSH stimulation, he developed abdominal mass sensation and incapacitating bone pain, with imaging confirming new and worsening metastatic lesions [22]. Despite rhTSH's benefits in avoiding severe hypothyroid symptoms, the case by Mejia and colleagues highlights the potential association with neoplastic progression [22].

One of the main hypotheses for the acute local reactions is that rhTSH induces inflammatory edema. This occurs as rhTSH may trigger vascular changes leading to increased blood flow and permeability in the tissues surrounding the tumor. The result is an inflammatory response that causes the tissues to swell, which might be perceived as an expansion of the tumor [21]. An alternative explanation is that although rhTSH is primarily used to stimulate iodine uptake into thyroid cells, it may also have a trophic effect, potentially leading to transient increases in tumor size due to increased cellular activity or proliferation [21, 23].

In patients with preexisting metastases, especially those located in critical areas like the central nervous system (CNS) or vertebral column, any increase in tissue volume, even from edema, can have important implications. It can cause compression of vital structures, leading to acute clinical symptoms such as pain, respiratory distress, or neurological deficits [23].

Our case demonstrates that it is likely a combination of factors leading to expansion of metastases of well-differentiated thyroid carcinoma following administration of rhTSH. Although periorbital edema and necrosis were present, it is unlikely that the patient's proptosis and intracranial expansion are entirely explained by edema alone.

Based on our case and other similar published cases, we recommend caution when using rhTSH in patients with known or suspected orbital or CNS metastases. If rhTSH is used for such lesions, adjunctive corticosteroid coverage should be considered. Luster et al [24] administered 8-mg dexamethasone, orally, twice daily, or 80-mg prednisone, orally, daily, to prevent tumor site edema before rhTSH in patients with brain or spinal metastases, with uneventful outcomes.

## Learning Points

- rhTSH can cause rapid enlargement of metastatic thyroid lesions, even those not previously evident. This can be especially consequential in the orbit or CNS.
- No specific guidelines exist for managing DTC with orbital metastases beyond surgery and RAI.
- Use rhTSH cautiously in patients with critical site metastases, currently or by history, and monitor closely for acute complications.

## Contributors

All authors made individual contributions to authorship. E.I. participated in the diagnosis of the patient, inpatient management, and obtained informed consent. L.K. and M.G. contributed to the diagnosis and direct management of the patient in the outpatient setting. T.G. performed the surgical procedures. M.M.A.H. supplied the histopathology images. M.E. reviewed and provided the radiological images. All authors contributed to the writing and review of the manuscript and approved the final draft.

## Data Availability

Data sharing is not applicable to this article as no datasets were generated or analyzed during the current study.

## Abbreviations

^18^F-FDG: 18F-fluorodeoxyglucose
CNS: central nervous system
CT: computed tomography
DTC: differentiated thyroid cancer
EBR: external beam radiation
FVPTC: follicular variant papillary thyroid carcinoma
I-131: iodine-131
L-T4: levothyroxine
MRI: magnetic resonance imaging
PET: positron emission tomography
PTC: papillary thyroid carcinoma
RAI: radioiodine
rhTSH: recombinant human thyrotropin
TSH: thyrotropin

## References

[1] Siegel RL, Kratzer TB, Giaquinto AN, Sung H, Jemal A. Cancer statistics, 2025. CA Cancer J Clin. 2025;75(1):10‐45. 39817679 10.3322/caac.21871 PMC11745215

[2] Carhill AA, Litofsky DR, Ross DS, et al Long-term outcomes following therapy in differentiated Thyroid Carcinoma: NTCTCS registry analysis 1987-2012. J Clin Endocrinol Metab. 2015;100(9):3270‐3279. 26171797 10.1210/JC.2015-1346 PMC5393522

[3] Kim H, Kim BH, Kim YK, et al Prevalence of BRAF(V600E) mutation in follicular variant of papillary Thyroid Carcinoma and non-invasive follicular Tumor with papillary-like nuclear features (NIFTP) in a BRAF(V600E) prevalent area. J Korean Med Sci. 2018;33(27):e75. 29962924 10.3346/jkms.2018.33.e75 PMC6021356

[4] Tang J, Kong D, Bu L, Wu G. Surgical management for follicular variant of papillary thyroid carcinoma. Oncotarget. 2017;8(45):79507‐79516. 29108330 10.18632/oncotarget.18525 PMC5668063

[5] Yu XM, Schneider DF, Leverson G, Chen H, Sippel RS. Follicular variant of papillary thyroid carcinoma is a unique clinical entity: a population-based study of 10,740 cases. Thyroid. 2013;23(10):1263‐1268. 23477346 10.1089/thy.2012.0453 PMC3787730

[6] Besic N, Luznik Z. Choroidal and orbital metastases from thyroid cancer. Thyroid. 2013;23(5):543‐551. 23082768 10.1089/thy.2012.0021

[7] Mahyuddin M, Theresia K, Anggraini N, Subekti HI. Orbital metastases as the initial clinical manifestation of thyroid carcinoma: a case series. Oman J Ophthalmol. 2022;15(1):85‐88. 35388250 10.4103/ojo.ojo_76_21 PMC8979405

[8] Pagsisihan DA, Aguilar AHI, Maningat MPDD. Orbital metastasis as initial manifestation of a widespread papillary thyroid microcarcinoma. BMJ Case Rep. 2015;2015:bcr2014208870. 10.1136/bcr-2014-208870 PMC4386322 25819821

[9] Ibrahim EY, Busaidy NL. Treatment and surveillance of advanced, metastatic iodine-resistant differentiated thyroid cancer. Curr Opin Oncol. 2017;29(2):151‐158. 28141684 10.1097/CCO.0000000000000349

[10] Gulec SA, Ahuja S, Avram AM, et al A joint statement from the American Thyroid Association, the European Association of Nuclear Medicine, the European Thyroid Association, the Society of Nuclear Medicine and Molecular Imaging on Current Diagnostic and Theranostic Approaches in the Management of Thyroid Cancer. Thyroid. 2021;31(7):1009‐1019. 33789450 10.1089/thy.2020.0826

[11] Klubo-Gwiezdzinska J, Burman KD, Van Nostrand D, Mete M, Jonklaas J, Wartofsky L. Potential use of recombinant human thyrotropin in the treatment of distant metastases in patients with differentiated thyroid cancer. Endocr Pract. 2013;19(1):139‐148. 23186979 10.4158/EP12244.RA PMC4185285

[12] Gomes-Lima CJ, Chittimoju S, Wehbeh L, et al Metastatic differentiated thyroid cancer survival is unaffected by mode of preparation for 131I administration. J Endocr Soc. 2022;6(5):bvac032. 35356009 10.1210/jendso/bvac032 PMC8962448

[13] Tuttle RM, Brokhin M, Omry G, et al Recombinant human TSH–assisted radioactive iodine remnant ablation achieves short-term clinical recurrence rates similar to those of traditional thyroid hormone withdrawal. J Nucl Med. 2008;49(5):764‐770. 18413378 10.2967/jnumed.107.049072

[14] Plyku D, Hobbs RF, Huang K, et al Recombinant human Thyroid-stimulating hormone versus Thyroid hormone withdrawal in (124)I PET/CT-based dosimetry for (131)I therapy of metastatic differentiated thyroid cancer. J Nucl Med. 2017;58(7):1146‐1154. 28104741 10.2967/jnumed.116.179366 PMC5493009

[15] Ma C, Xie J, Liu W, et al Recombinant human thyrotropin (rhTSH) aided radioiodine treatment for residual or metastatic differentiated thyroid cancer. Cochrane Database Syst Rev. 2010;2010(11):Cd008302. 21069705 10.1002/14651858.CD008302.pub2 PMC6718234

[16] Wondisford FE, Usala SJ, DeCherney GS, et al Cloning of the human thyrotropin beta-subunit gene and transient expression of biologically active human thyrotropin after gene transfection. Mol Endocrinol. 1988;2(1):32‐39. 3398841 10.1210/mend-2-1-32

[17] Taïeb D, Sebag F, Cherenko M, et al Quality of life changes and clinical outcomes in thyroid cancer patients undergoing radioiodine remnant ablation (RRA) with recombinant human TSH (rhTSH): a randomized controlled study. Clin Endocrinol (Oxf). 2009;71(1):115‐123. 18803678 10.1111/j.1365-2265.2008.03424.x

[18] Haugen BR, Cooper DS, Emerson CH, et al Expanding indications for recombinant human TSH in thyroid cancer. Thyroid. 2008;18(7):687‐694. 18630995 10.1089/thy.2008.0162 PMC2637556

[19] Campennì A, Ruggeri RM, Siracusa M, et al Early preablation rhTSH-stimulated thyroglobulin predicts outcome of differentiated thyroid cancer (DTC) patients. Eur J Nucl Med Mol Imaging. 2021;48(8):2466‐2475. 33416957 10.1007/s00259-020-05153-7

[20] Chen MK, Doddamane I, Cheng DW. Recombinant human thyroid-stimulating hormone as an alternative for thyroid hormone withdrawal in thyroid cancer management. Curr Opin Oncol. 2010;22(1):6‐10. 19844179 10.1097/CCO.0b013e3283339d5d

[21] Braga M, Ringel MD, Cooper DS. Sudden enlargement of local recurrent thyroid tumor after recombinant human TSH administration. J Clin Endocrinol Metab. 2001;86(11):5148‐5151. 11701668 10.1210/jcem.86.11.8055

[22] Mejia MG, de Bogotá C, José HS, Veronesi LA, Concha DC. Relationship between rhTSH administration and thyroid cancer progression: a case report. J Endocr Soc. 2021;5(Suppl 1):A899‐A899.

[23] Vargas GE, Uy H, Bazan C, Guise TA, Bruder JM. Hemiplegia after thyrotropin alfa in a hypothyroid patient with thyroid carcinoma metastatic to the brain. J Clin Endocrinol Metab. 1999;84(11):3867‐3871. 10566621 10.1210/jcem.84.11.6161

[24] Luster M, Lassmann M, Haenscheid H, Michalowski U, Incerti C, Reiners C. Use of recombinant human thyrotropin before radioiodine therapy in patients with advanced differentiated thyroid carcinoma. J Clin Endocrinol Metab. 2000;85(10):3640‐3645. 11061516 10.1210/jcem.85.10.6903

